# Blockchain-Modeled Edge-Computing-Based Smart Home Monitoring System with Energy Usage Prediction

**DOI:** 10.3390/s23115263

**Published:** 2023-06-01

**Authors:** Faiza Iqbal, Ayesha Altaf, Zeest Waris, Daniel Gavilanes Aray, Miguel Angel López Flores, Isabel de la Torre Díez, Imran Ashraf

**Affiliations:** 1 Department of Computer Science, University of Engineering & Technology (UET), Lahore 54890, Pakistan; faiza.iqbal@uet.edu.pk (F.I.); 2022phdcs7@student.uet.edu.pk (Z.W.); 2 Research Group on Foods, Universidad Europea del Atlántico, Isabel Torres 21, 39011 Santander, Spain; daniel.gavilanes@uneatlantico.es (D.G.A.); miguelangel.lopez@uneatlantico.es (M.A.L.F.); 3 Research Group on Foods, Universidad Internacional Iberoamericana Arecibo, Arecibo, PR 00613, USA; 4 Universidade Internacional do Cuanza, Cuito EN250, Bié, Angola; 5 Universidad Internacional Iberoamericana, Campeche 24560, Mexico; 6 Instituto Politécnico Nacional, UPIICSA, Ciudad de México 04510, Mexico; 7 Department of Signal Theory, Communications and Telematics Engineering, Unviersity of Valladolid, Paseo de Belén, 15, 47011 Valladolid, Spain; isator@tel.uva.es; 8 Department of Information and Communication Engineering, Yeungnam University, Gyeongsan 38541, Republic of Korea

**Keywords:** Internet of Things, blockchain, edge computing, privacy, machine learning, net-metering

## Abstract

Internet of Things (IoT) has made significant strides in energy management systems recently. Due to the continually increasing cost of energy, supply–demand disparities, and rising carbon footprints, the need for smart homes for monitoring, managing, and conserving energy has increased. In IoT-based systems, device data are delivered to the network edge before being stored in the fog or cloud for further transactions. This raises worries about the data’s security, privacy, and veracity. It is vital to monitor who accesses and updates this information to protect IoT end-users linked to IoT devices. Smart meters are installed in smart homes and are susceptible to numerous cyber attacks. Access to IoT devices and related data must be secured to prevent misuse and protect IoT users’ privacy. The purpose of this research was to design a blockchain-based edge computing method for securing the smart home system, in conjunction with machine learning techniques, in order to construct a secure smart home system with energy usage prediction and user profiling. The research proposes a blockchain-based smart home system that can continuously monitor IoT-enabled smart home appliances such as smart microwaves, dishwashers, furnaces, and refrigerators, among others. An approach based on machine learning was utilized to train the auto-regressive integrated moving average (ARIMA) model for energy usage prediction, which is provided in the user’s wallet, to estimate energy consumption and maintain user profiles. The model was tested using the moving average statistical model, the ARIMA model, and the deep-learning-based long short-term memory (LSTM) model on a dataset of smart-home-based energy usage under changing weather conditions. The findings of the analysis reveal that the LSTM model accurately forecasts the energy usage of smart homes.

## 1. Introduction

The Internet of Things (IoT) connects the digital and physical worlds. It has shown significant growth over the past decade [1]. The embedded smart heterogeneous devices and related technologies have grown due to the quick development of IoT. For an application to function, a number of heterogeneous and diverse protocols and resource-constrained devices must interact and synchronize with one another under the IoT paradigm [2,3]. IoT applications are usually based on real-time monitoring techniques. These limitations specifically affect interoperability and security, creating hindrances in IoT services. Due to growing problems with environmental pollution and global energy depletion, energy management is required as a result of rising energy prices brought on by a variety of variables, including supply and demand, weather forecasts, global markets, and governmental regulations [4]. The smart home system obtains utility pricing data from smart meters by utilizing the advanced metering infrastructure (AMI). Smart meters can monitor and regulate electrical devices in addition to measuring power usage [5]. Despite the smart home concept becoming more and more popular, such technology is susceptible to numerous security risks. Data transmission using wireless technology greatly decreases the need for physical labor; however, the smart home network community is susceptible to data theft [5]. Technical advancements have been made in creating frameworks for the identification and mitigation of these costly assaults [6]. Smart meters show which appliances are now consuming electricity, how much they cost, and what they are. This knowledge can be abused by many different kinds of people. The unauthorized use of personal data constitutes a privacy infringement and might have very serious repercussions [7].

According to the report “State of IoT Security” a 22% increase has been observed in cyber attacks on IoT applications. In addition, such attacks being highly graded and sophisticated indicate serious concerns [8,9]. As devices have limited processing and storage capabilities, smart devices are vulnerable to assaults as these devices violate many policies regarding security [10,11]. Most fundamental security aspects such as confidentiality, integrity, and availability must be preserved by every application [12] but, due to real-time monitoring and extensive applications in the IoT paradigm, tens of thousands of devices are embedded and connected, creating trouble for the present server–client model in synchronization and interoperability. Specifically, in smart homes, a variety of IoT-enabled devices are being deployed from various vendors [13]. The immutability of data, system transparency, transactional security, transparency, cryptographic protection, and other distinctive aspects of blockchain enabled it to advance a variety of technologies, including voting processes, IoT applications, supply chain management, finance, healthcare, and insurance, among others. Blockchain development was accelerated by the growing desire for technical advancements. Financial transactions were made possible without relying on middleware due to a peer-to-peer electronic cash system such as Bitcoin [14]. For the elimination of third-party involvement, cryptography is used. For IoT, there are numerous methods for accomplishing confidentiality and security [15].

Various studies are intrigued by the integration of blockchain into IoT biological networks. Only two or three studies, however, have looked into how blockchain supports achieving IoT security requirements. In this area, we describe the projects that describe such partner-level organizations and display the phenomena of projects to satisfy security needs. Since the majority of the work focuses on using blockchain advances to achieve IoT benefits, IoT security analysis is tiresome and the blockchain is constrained. However, with the advancement in Web 3.0, the world is moving toward blockchain-modeled systems for securing data and transactions. In the IoT, a large number of integrated sensors and actuators are in communication with one another and carry out transactions for each action. Most of the time, third parties are required to complete online transactions. This not only imposes restrictions on the transaction’s minimum allowable cost but also increases the risk of intrusion and data misuse [16].

There are two pricing structures. The first is where the utility forecasts the future price of electricity and is used to facilitate the scheduling of smart homes, and real-time pricing is utilized to bill the consumers depending on the energy usage during the previous time. By carrying this out, the energy burden on the electricity system is balanced. Home-level distributed generation leverages the local resource to directly supply energy to clients, lessening the strain of power generation and transmission. Additionally, clients are urged to sell excess renewable energy back to the power grid to receive rewards. Various machine learning algorithms are used to predict the future energy usage of the users on the previous trend so that a relevant amount of energy can be purchased from the grid. The installment of smart meters controls and monitors the energy usage of various smart home appliances and offloads the burden in peak hours in order to adjust according to energy purchase and to make the user’s data secure from theft and misuse.

The problem statement of this research is thus defined as designing a blockchain-modeled smart home monitoring system for securing against data misuse and providing energy usage prediction for cost estimation for the smart home user under different weather conditions using machine learning. This research presented a blockchain-based smart home system to continuously monitor IoT-enabled smart home devices such as smart microwaves, dishwashers, furnaces, fridges, etc. A machine-learning-based approach was applied to train the auto-regressive integrated moving average (ARIMA) model for energy usage prediction, which is displayed in the user gadgets. The future energy usage prediction may then be used for cost estimation or energy buying or selling as the practical implementation.

The contribution of this research work is twofold: first, to present the secure smart home system based on the blockchain model and, second, to estimate the energy usage of the smart home system based on weather conditions to estimate the future energy usage of the smart homes.

The rest of the paper comprises four sections. The literature review is presented in Section 2 whereas materials and methods are discussed in Section 3. Section 4 provides experimental results and discussions. Finally, the conclusion is given in Section 5.

## 2. Literature Review

Blockchain is one of the ways to strengthen IoT security, especially in distributed systems, and many studies support the use of blockchain-based IoT systems and secure transactions [17]. Blockchain technology is based on the distributed ledger against the centralized database. All the participating entities in the transaction have the ledger copy. In the IoT paradigm where a large amount of transaction data are involved, the disfunction of centralized database results in a loss of data. The blockchain can resolve this issue, in addition to ensuring the transparency of transactions. Due to these promising features, blockchain is gaining attraction for a wide range of applications, including IoT.

Numerous studies have proved the security-enhancing potential of blockchain technology in a variety of fields [18,19,20,21]. Another study [22] proposed a verifiable query layer (VQL) to address blockchain data querying inefficiencies and validity. While blockchain technology has found expanding uses, direct inquiries are time-consuming and indirect queries are compromised, making practical deployment difficult. Another study [23] proposed vChain+ to improve blockchain database search. Blockchain technology, popularized by cryptocurrencies and decentralized applications, is safe and tamper-resistant. The study criticized vChain’s worst-case linear-scan search performance and unrealistic public key management. vChain+, an upgraded searchable blockchain system, overcomes these restrictions while providing efficient verified boolean range queries and adding capabilities.

Balogh et al. [4] used the application layer, network layer, and physical layer to make the basic architecture of the IoT systems. Many devices are embedded in the physical layer and interlinked through the gateway. These hardware devices have restricted potential exposure to the assailant. In such a scenario, replacing each affected module is not possible; hence, a mechanism is needed to tackle such problems as network layer attacks and application layer attacks. Many issues and related challenges are linked with IoT interoperability and security [4]. Similarly, the study [24] identified that IoT/CPS systems are not understood completely relative to traditional ones, as these are widely distributed in an uncontrolled manner. The IoT environment is an amalgamation of heterogeneous technologies, various protocols, and processes. Moreover, in IoT systems, there is no standard, stable architecture, nor security mechanism present to integrate different systems. Consequently, varied addressing formats and models introduce complexity in IoT systems, creating the issue of interoperability. Another concern in the IoT environment is the presence of nodal platforms with controlled electrical power, limited memory, and low computational power, which are therefore incapable of incorporating heavy firewalls, ultimately giving rise to security concerns [24].

The study in [25] provided a detailed aspect of the vulnerabilities and thus security weaknesses found in each IoT layer and offered blockchain-enabled solutions. Cybersecurity aims at providing confidentiality, integrity, and authentication as three main aspects of tackling cyber attacks and ensuring the protection of cyber–physical systems. In the context of IoT, confidentiality indicates that data packets are not seized and peeked into, or that the host is not compromised so that an unauthorized person can attain sensitive data, information, or credentials. Integrity ensures that data received or sent are not modified in any unauthorized way. Availability is about all the modules in the system that are working properly and are not prohibited from proper functioning in case the module is infected by some malicious agent or intrusion. It thus ensures disinfecting the device immediately and not operating in a compromised way [25].

The authors in [26] worked on factors improving security to ensure CIA goals. To strengthen security, it is recommended to divide IoT devices into those that need direct Internet connectivity into network segments and those that should be forbidden network access. The network segment should be kept under monitoring for any potential unexpected traffic and be subjected to the necessary response [26]. The study in [8] focused on efficient message filtering for privacy-preserving authentication and secure packet forwarding for aggregated transmission evidence generation. Privacy of identification, location, input, output privacy, and function privacy is required for IoT security. Secure IoT cloud systems are dependent on trustworthiness, resiliency, language, coding method, data validation and handling, and dependability. Another study [27] presents an approach where digital identity management is enabled by blockchain. Consequently, there is no need for a central authority and blockchain can be enabled to hide or share IDs. Blockchain implements distributed authority and creates secure layers to avoid record tampering. In the Metaverse, the digital world is relying on blockchain technology to solve confidentiality, integrity, and authentication-related problems [25].

The authors in [9] discussed different modules and their functionality present in smart homes [28,29] and outlined the transactional methods associated with the smart home. They also proposed the metrics for efficiency in terms of processing time traffic and energy consumption. Integrity, confidentiality, and availability are ensured as security goals. The study in [15] proposed an Ethereum-based distributed smart contract in replace of the orthodox centralized system to tackle DDoS attacks. This was achieved by giving resources to each device and enabling the proposed system to distinguish between untrusted and trusted devices.

The study in [30] investigated the vulnerability aspects of IoT networks regarding DDoS attacks. Furthermore, how they affect the services, blockchain methods for tackles DDoS attacks, and the challenges in integrating IoT and blockchain were also analyzed. In [31], the authors developed an algorithm that mines consumer behavior data exclusively and applied machine learning models to advise activities for optimal energy consumption at homes. The algorithm may be utilized for energy optimization in smart homes without reducing the comfort of the occupants. The digital-STROM home automation system’s event data are analyzed by the system for recurring and periodic patterns. These patterns are transformed into association rules, given a priority order, and compared to the inhabitants’ recent behavior. The system makes a recommendation to the occupants if it finds ways to conserve energy without reducing comfort. An assortment of test homes were placed under the system’s deployment. The test subjects had the option of rating how the advice affected their level of comfort. During a second test phase, the system’s parameters were modified based on this feedback to increase accuracy [31].

For resource-constrained IoT devices, a high computing power is needed. Cryptographic techniques in blockchain consume higher power, which is a big challenge for blockchain technology. For blockchain to offer the appropriate level of security and anonymity, asymmetric encryption methods are crucial [32]. PoS has been regarded as more secure and energy-efficient than PoW. The Ethereum platform, which is a well-known blockchain application, uses PoS. Ethereum is the first platform to make the use of smart contracts possible. The Ethash function is used by Ethereum-based coins to implement the Keccak hash function [32]. Table 1 lists and analyze existing literature in terms of IoT plateform, smart home energy, blockchain plateform and security features.

## 3. Materials and Methods

**Figure 1:**
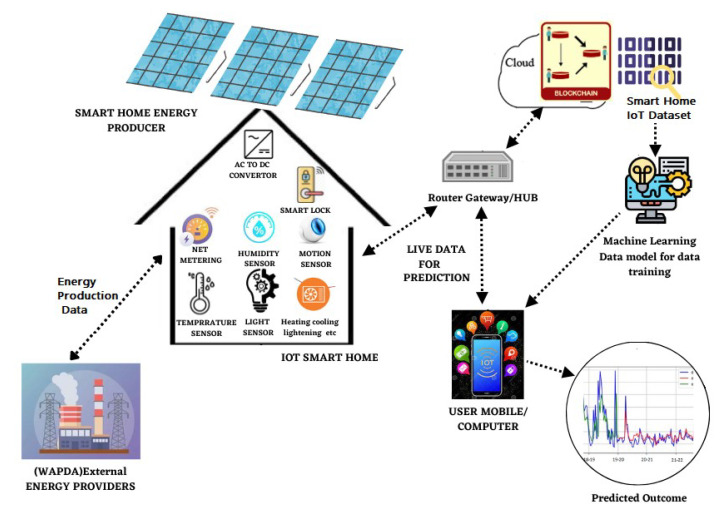
Proposed smart home paradigm for forecasting of energy consumption, energy management, and net metering.

The current study utilized the IoT concept of smart homes together with blockchain technology to build an efficient and secure system. A smart home scenario was set up with IoT, energy, and weather-data-based machine learning, prediction, and blockchain modeling for securing the data as shown in Figure 1. The smart home consists of smart appliances such as microwaves, dishwashers, furnaces, fridges, air condition, ovens, television, etc., and IoT-enabled sensors to measure environmental factors such as temperature, humidity, visibility, pressure, and weather conditions such as cloud cover, wind speed, dew point, etc. The smart homework uses the demand response concept, where the consumer can adapt and alter their electricity usage for a limited time in response to time-based financial incentives when the power supply is limited or electrical networks are overloaded.

A smart grid is present in the system to intelligently monitor and regulate electricity from all energy-producing sources and to provide and purchase energy based on consumer demand. Two sources provide the electricity that smart gadgets and sensors require to function. The consumer purchases prepaid electricity from the energy/electricity provider, and the smart home’s solar panels produce energy for consumption as well. The concept of net metering was utilized in this circumstance. Installing smart meters made net metering possible by monitoring families’ energy demand and consumption in real-time, thus accurately predicting the energy demand and supply of the smart home.

The smart home consumes energy for the benefit of service providers and provides the excess energy generated by solar panels to powerhouses. The installed sensors measure the energy consumption of the appliances, as well as the weather conditions, since electricity consumption is assessed in association with weather conditions so that the necessary amount of electricity may be purchased or sold from and to the grid. The history and present energy consumption of home appliances is given to a machine learning model for data training in order to forecast home appliance energy consumption.

A user interface and notification system were specifically incorporated into the system to enable efficient communication with customers regarding time-based financial incentives for demand response. Consumers interact with the system through the user interface, which gives them real-time information and notifications so that they can make wise decisions. The home energy management systems’ applications serve as the user interface for all individual devices reporting the visual feedback of energy consumption and need to the consumer. Through this interface, users can easily access and monitor their energy usage statistics, real-time energy costs, and information on time-based financial incentives for demand response. The notification system also alerts consumers to time-based financial incentives. It uses push notifications, email alerts, SMS messaging, and smart home device audio notifications. Users can personalize their notifications and communication channels based on incentive urgency. The technology alerts users of time-based financial incentives. This enables users to actively participate in demand response programs and make appropriate adjustments to their energy consumption habits. The administrator/user accesses the application, views the sensor data, and controls the appliances through the smartphone/tablet/computer, etc.

The salient features of this research work are described below.

- To secure the IoT smart home system, a blockchain model was used that keeps track of each device’s credentials and authenticates its access and usage.
- A blockchain-based record-keeping system was designed for storing data such as those generated from intelligent smart home devices and equipment.
- In the proposed model, the blockchain-based record-keeping system was developed in a layered approach to keep data copies at the cloud levels.
- For data security, IoT device registration services were modeled. An encryption–decryption method was applied to data transactions. SHA-256 hash algorithm was used for blockchain address mining.
- A machine-learning-based critical alerts prediction system was trained from the smart home dataset available at [26].
- A blockchain-modeled smart home system was designed to continuously monitor IoT-enabled smart home devices such as smart microwaves, dishwashers, furnaces, and fridges. The energy usage prediction is displayed in the user’s wallet.
- Machine learning algorithms were applied to learn the critical situation from the energy usage monitoring database to produce the alerts.

The proposed smart home monitoring system was built on the Internet of Things (IoT), which links numerous appliances and sensors located throughout the house. These IoT gadgets gather real-time data on user behavior, ambient factors, and energy use. They safely send these data to the hub of our system, where they are handled and examined. Algorithms for machine learning were used to analyze the gathered data and derive valuable insights. These algorithms can analyze abnormalities, spot patterns and trends in energy usage, and forecast future energy consumption using data from the past. Our technology can anticipate energy usage accurately and provide proactive recommendations for maximizing energy efficiency by continuously learning from the data.

By incorporating blockchain technology, the system guarantees the immutability, transparency, and security of the data kept there. This makes it possible to build a decentralized, impenetrable ledger where financial transactions, demand response incentives, and data on energy usage can all be recorded and validated. The blockchain also enables secure peer-to-peer communication without the use of middlemen between various groups, including homeowners, utility providers, and outside service providers.

Before sending data to the central hub, our system processes and analyzes them locally at edge devices or gateways using edge computing technology. This distributed strategy decreases the requirement for massive data transmission to faraway servers while lowering latency and improving real-time responsiveness. The system’s ability to handle a high number of connected devices and effectively manage data processing and storage resources is made possible by edge computing, which also helps the system’s scalability.

The technology meets the overall objective of a secure and effective smart home by merging these elements. Blockchain offers data confidentiality and transparency, IoT devices supply real-time data, machine learning algorithms assess and forecast energy usage, and edge computing improves system performance and scalability. Together, they give homes the ability to make knowledgeable decisions, optimize energy use, take part in demand response programs, and make a difference in the development of more sustainable and affordable energy management.

Smart contracts are a key part of our blockchain-based, edge-computing-based smart home monitoring system. They help to make sure that payments are accurate and safe while also being transparent and automated. Here is a detailed look at how smart contracts are defined and managed in the system:

What are “smart contracts”? Smart contracts are agreements that carry out themselves based on rules and conditions that have already been set. Smart contracts in our system are written in a programming language that works with the blockchain platform we use (e.g., Solidity for Ethereum). These contracts include the specific terms and conditions of the financial agreements about incentives and payments for responding to demand.

Integration with Blockchain: Smart contracts are deployed and run on the blockchain, which is a decentralized, unchangeable ledger that records and verifies transactions transparently. The blockchain platform makes sure that smart contracts are carried out in a way that is secure, clear, and hard to change.

Accuracy of Payments: To make sure payments are correct, smart contracts in our system use data from IoT devices that show how much energy is being used in real-time. These devices give accurate readings of how much energy each appliance or the whole house uses. Smart contracts use this information to figure out the payments based on formulas or rates that were already agreed upon in the contract.

Secure Payment Execution: Payments that are made through smart contracts are made to be safe and impossible to change. When certain conditions are met, such as reducing energy use by a certain amount during a demand response event, smart contracts automatically start the payment process. The amount of the payment is based on the terms that were agreed upon, which could include the amount of energy saved, the length of participation, or any other set criteria.

Auditing: One of the best things about using blockchain technology is that it is transparent by nature. The blockchain ledger keeps track of all transactions, including payments made through smart contracts. This makes it easy to carry out audits and check that payments were made correctly. Anyone who is allowed to be part of the system, such as homeowners, utility companies, or government agencies, can access the blockchain and check the details of the payment.

Security Measures: To make sure that payments are safe, our system uses cryptographic techniques and consensus mechanisms from the blockchain platform that it is built on. These steps make sure that payment information stored on the blockchain cannot be accessed, changed, or tampered with by people who should not be able to. The payment process is even safer when public-key cryptography and consensus algorithms (such as proof-of-work or proof-of-stake) are used. The proposed system makes sure that payments for demand response incentives are made correctly and safely by using smart contracts on the blockchain. The integration of real-time data about energy use, the transparency of the blockchain ledger, and the automation of smart contracts all help to make the payment process reliable and auditable.

**Figure 2:**
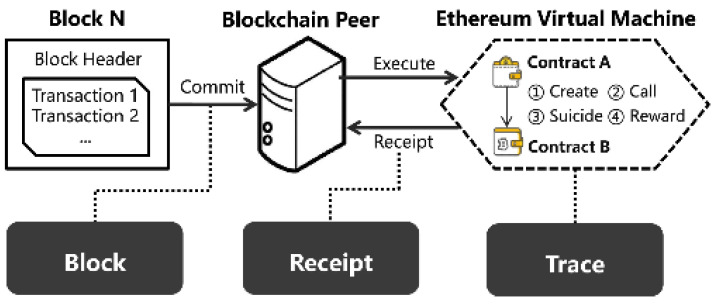
Ethereum virtual machine executing smart contracts for blockchain peer distributed workflow.

A workflow for smart contract execution on Ethereum virtual machine for peer-distributed blockchain is presented in Figure 2. In the proposed system, the blockchain keeps a record of the user energy profile, and ether-coins transactions are performed by smart contracts. The Ethereum virtual machine traces the execution of smart contracts between smart home users and energy providers under the net-metering concept. The user energy usage profile, net-metering data, and ether wallet payments are kept secret in the blockchain.

Any change in the data by any party is recorded into the distributed ledger blockchain, which applies SHA-256 hashing for this purpose. The data can be viewed by the user and authorized persons only. If any party intruder tries to temper the transaction record or data, it is compared with the data of every block, and if found tempered, it is discarded, thus ensuring data security. The payment is automatically made using the Ethereum services from user to producer wallets as per defined smart contracts. The proposed system employs the ARIMA model for prediction and the following factors are considered for ARIMA.

### 3.1. ARIMA

In the proposed blockchain-modeled edge-computing-based smart home monitoring system with energy usage prediction, the ARIMA model is a useful forecasting tool, but certain circumstances can affect its accuracy. The method accounts for and addresses these factors as follows.

#### 3.1.1. Feature Engineering

We identified and added relevant factors to the model to improve prediction accuracy. Historical energy consumption statistics, weather, occupancy, time of day, and other contextual factors may affect energy usage patterns. To increase prediction accuracy, we considered these criteria.

#### 3.1.2. Data Preprocessing

To avoid accuracy difficulties, we preprocessed data before applying the ARIMA model. This included processing missing or incorrect data, adjusting or scaling variables, and addressing outliers and anomalies. We minimized noise and disruptions that could affect prediction performance by guaranteeing data quality and cleanliness.

#### 3.1.3. Model Selection and Evaluation

ARIMA is a popular time series forecasting model, although it may not capture all energy usage trends. We selected and evaluated models to overcome this constraint. This involved comparing SARIMA, exponential smoothing, and machine-learning-based forecasting models against ARIMA. We tested different models to find the optimal one for our application and evaluated unaccounted aspects.

#### 3.1.4. Sensitivity Analysis

We examined our forecasts’ sensitivity to unexplained factors. These analyses tested ARIMA model predictions by perturbing input variables or simulating situations. We examined the model’s response to these alterations to find hidden constraints or uncertainty.

#### 3.1.5. Continuous Improvement

No model can capture all energy usage parameters. Our system design includes ongoing improvement. This method comprises evaluating the model’s performance, collecting user input, and adding new factors or upgrading forecasting algorithms based on fresh research. We can enhance our forecasts by iterating.

### 3.2. Dataset and Processing

For training of machine learning models, this research work employed the smart home dataset available at Kaggle for IoT-based applications [43]. The dataset contains the readings of a smart meter for a time span of 1 min. The dataset includes weather data for that location, as well as readings of household appliances in kW taken every minute from a smart meter. The dataset has 32 parameters for which data are logged. The parameters include time, generation (kW), use (kW), house overall (kW), home office (kW), furnace 1 (kW), furnace 2 (kW), garage door (kW), refrigerator (kW), dishwasher (kW), wine cellar (kW), kitchens 12 (kW), 14 (kW), 38 (kW), well (kW), barn (kW), living room (kW), microwave (kW), solar (kW), temperature, humidity, icon, apparent, summary, visibility pressure, cloud cover and blow bearing temperature, wind tempo, precipitation, dew point, intensity, and rainfall. Out of these 32 parameters, 19 parameters are about energy usage and production, whereas the remaining 13 parameters store information about weather stats.

**Figure 3:**
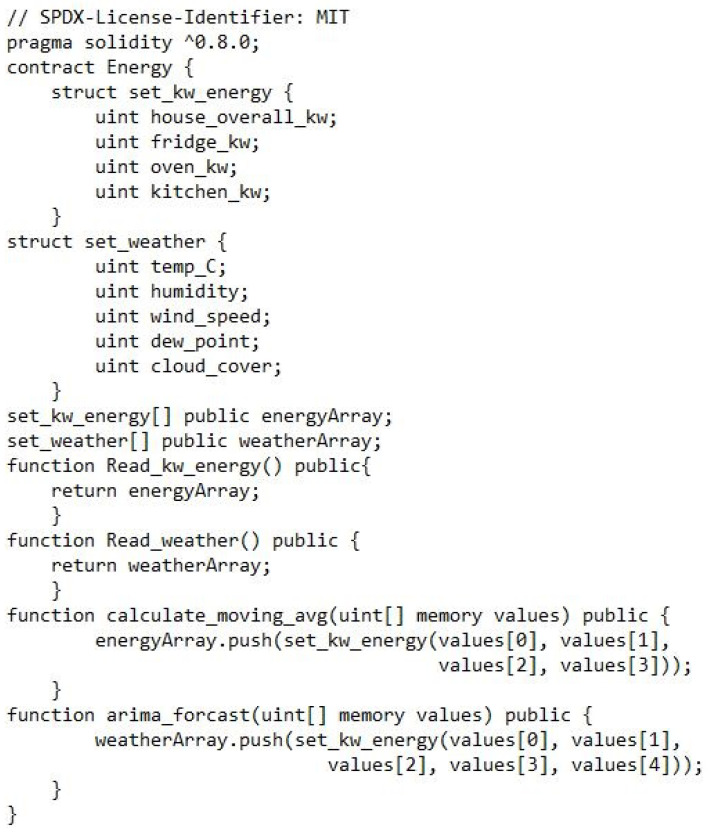
Blockchain code segment.

The blockchain code segment is shown in Figure 3, which we used in this study as a baseline smart contract in solidity blockchain language to make the proposed model work.

This is a solidity smart contract that defines an “energy” contract. The contract has a few different functions and data structures.

- The set_kw_energy struct defines a set of energy values for a specific house, including the overall kW, kW for the fridge, oven, and kitchen;
- The set_weather struct defines a set of weather values, including temperature in Celsius, humidity, wind speed, dew point, and cloud cover.
- The energyArray and weatherArray are arrays that store sets of energy and weather values, respectively.
- The Read_kw_energy function is a public function that returns the energyArray array, allowing users to read the stored energy data.
- The Read_weather function is a public function that returns the weatherArray array, allowing users to read the stored weather data.
- The calculate_moving_avg function is a public function that takes an array of four unit values (for overall kW, fridge kW, oven kW, and kitchen kW) and adds them to the energyArray array as a new set_kw_energy object.
- The arima_forcast function is a public function that takes an array of five unit values (for temperature in Celsius, humidity, wind speed, dew point, and cloud cover) and adds them to the weatherArray array as a new set_weather object.

**Figure 4:**
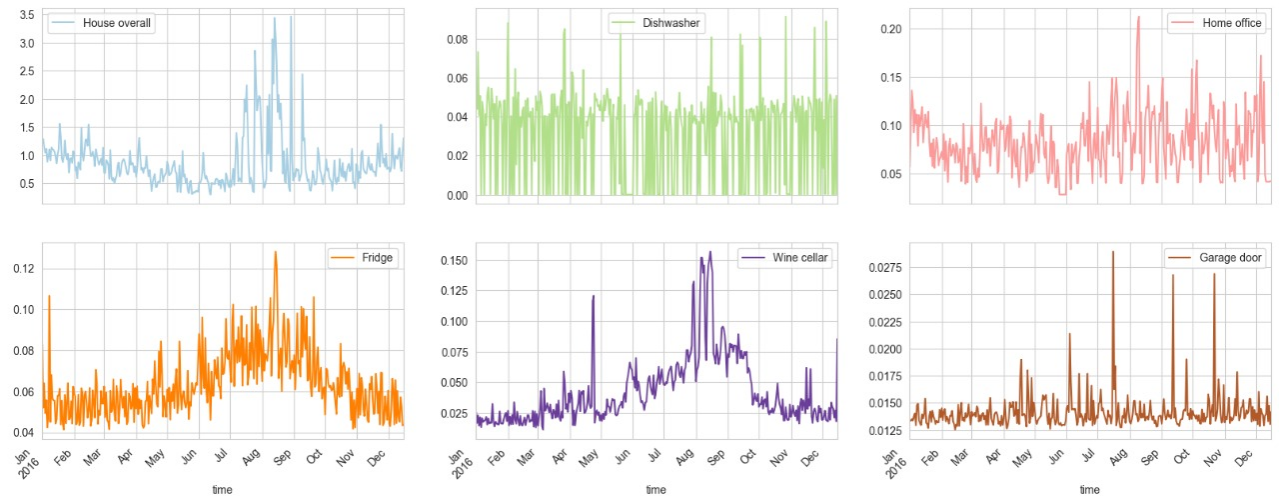
First 6 energy columns (resampled by day) show house overall energy consumption is at peak in July, August, and September.

For visualization purposes, we plotted each of these columns as graphs. The first 6 energy columns (resampled by day) are plotted in Figure 4. It shows that the house’s overall energy consumption is at its peak in July, August, and September. The energy consumption of the dishwasher is almost constant throughout the year, except for some specific times when it is used more than the average. The peak energy consumption in the said months is determined to be because of the fridge, wine cellar, and home office.

**Figure 5:**
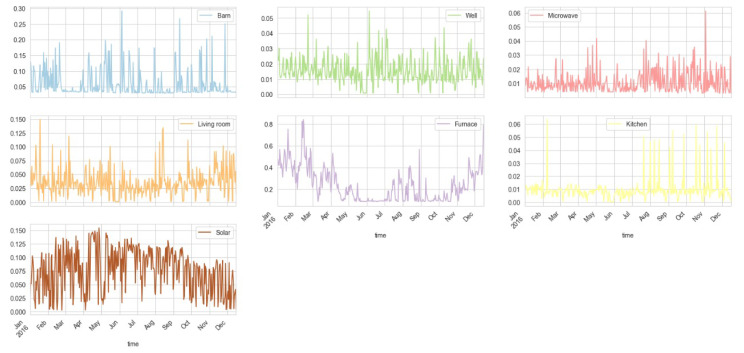
Rest (9) of the energy columns (resampled by day) shows the use of energy by different equipment.

The rest of the energy columns (resampled by day) are plotted in Figure 5. The use of furnaces is at its maximum in the months from November to February, which shows that the region is colder in these months. The use of energy in the living room, by the well, in the barn, and in the microwave varies around average except for some specific events. We may consider that use of energy by this equipment does not vary throughout the year. Out of the total 19 energy parameters, we have a total of 13 parameters that are related to the usage of energy, whereas the remaining 6 energy parameters are related to energy production.

**Figure 6:**
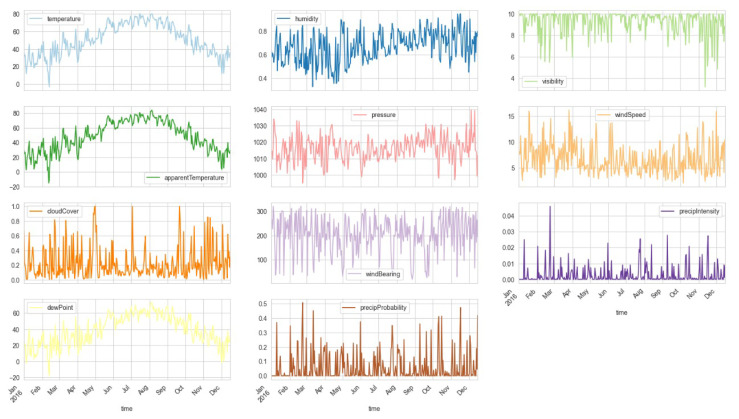
Visualization of weather columns (resampled by day) for a full year on a monthly basis. It presents the variation in temperature, humidity, visibility, etc., throughout the year.

For visualization of 11 weather parameters, the plots are presented in Figure 6 for a full year on a monthly basis. It presents the variation in temperature, humidity, visibility, etc., throughout the year.

We preprocessed the dataset to determine the energy correlation from the dataset to identify the usefulness of each parameter. The correlation coefficient is a statistical measure that tells how strong and in which direction two variables are linked in a straight line. The scale goes from −1 to +1, and each number represents a different level of correlation strength. Variables with a correlation coefficient near +1 are strongly correlated. As one variable rises, the other rises correspondingly. A number of +1 indicates that energy consumption increases at a constant rate when the outdoor temperature rises. The positive correlation increases as the correlation coefficient approaches +1. No linear correlation exists when the correlation coefficient is close to 0. If the correlation coefficient is around 0, changes in one variable do not correlate with changes in the other. If we are studying the correlation between energy usage and the number of bedrooms in a house, a result close to 0 indicates no meaningful link. A correlation coefficient around −1 indicates a strong negative association. One variable grows, and the other decreases correspondingly. For example, a score of −1 would indicate that energy use drops consistently as power prices rise. The variables’ negative connection increases as the correlation coefficient approaches −1.

**Figure 7:**
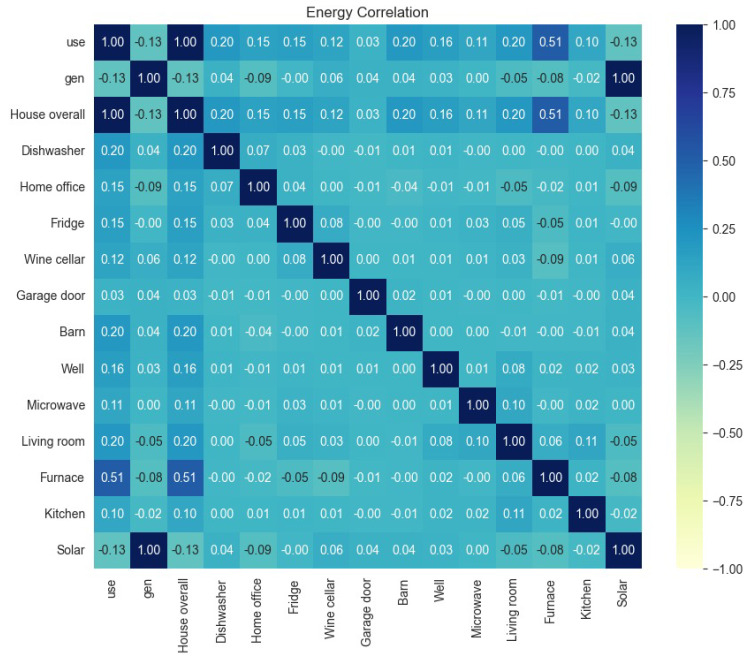
Energy correlation of smart home dataset with weather information to identify the usefulness of each parameter. The heat bar shown on the right side of the heat map presents a scale from −1 to +1.

The energy correlation information is presented in Figure 7. The heat bar shown on the right side of the heat map presents a scale from −1 to +1. The blue color and its neighborhood present less correlation among the parameters highlighted in these colors inside the heat map. The dark blue color range presents a positive correlation among the parameters highlighted in these colors inside the heat map. The whitish-blue color represents a kind of neutralism for the correlation between the weather parameters. The highest correlation is observed between solar and generation. The second highest correlation exists between furnaces and house overall usage. There is no negative correlation in the given data set.

**Figure 8:**
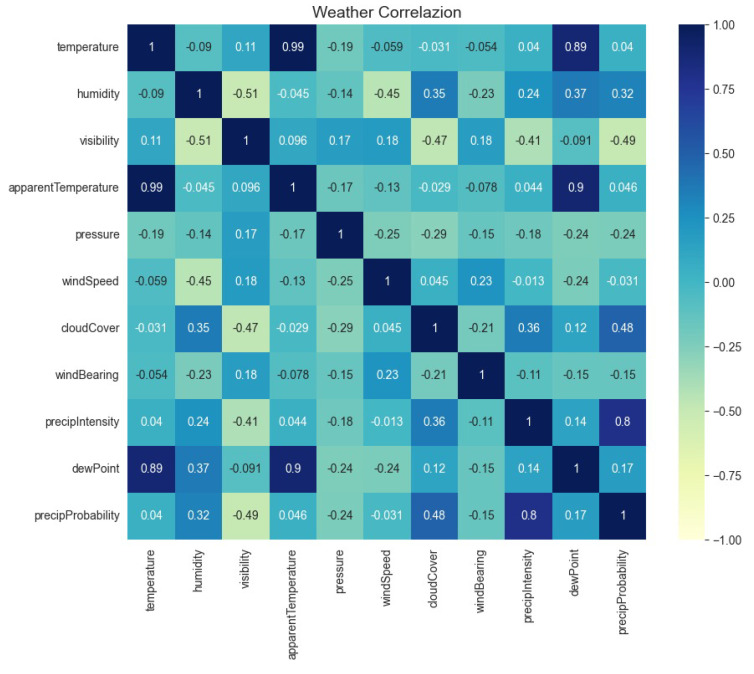
Correlation of weather parameters for the smart home.

Next, the dataset was preprocessed to check correlations of weather-related features only. The outcome is presented as a heatmap in Figure 8.

A scale from −1 to +1 is presented in the heat bar on the right side of the heat map. The characteristics highlighted in these colors inside the heat map show the last link with the blue color and its surroundings. The heat map’s orange color range shows a positive link between the parameters that are highlighted in these colors. The black color symbolizes a form of neutrality for the correlation of meteorological parameters. A high correlation is observed between temperature and dew point, precipitation intensity, and precipitation probability. At the next low level, humidity correlates with cloud cover, precipitation intensity, and precipitation probability.

### 3.3. Blockchain-Based Security Features

In the smart home monitoring system that is based on blockchain and edge computing, we built in a number of security measures to make sure that the system is confidential, integral, and robust. In particular, these parts of blockchain technology were used:

- Distributed Ledger: The system uses a distributed ledger, which is a key part of blockchain technology, to store information in a way that is not controlled by a single entity. This gets rid of the need for a central authority or a single point of failure. This makes the system more resistant to attacks and data breaches.
- Data that cannot be changed: Once the data are on the blockchain, they cannot be changed or tampered with. This keeps the data from being changed by people who should not be able to. Each transaction or change to the system is saved as a block, which is cryptographically linked to the blocks that came before it. This creates a chain of transactions that cannot be changed after the fact.
- Consensus Mechanisms: Blockchain uses consensus mechanisms to make sure that everyone in the network agrees on the validity of transactions and the order in which they are added to the blockchain. Proof-of-work (PoW) and proof-of-stake (PoS) are two common consensus mechanisms. They protect against bad behavior by requiring participants to solve computational puzzles or stake their own tokens.
- Cryptographic Security: To protect data and transactions, blockchain technology uses cryptography. Public-key cryptography makes sure that communication and authentication are secure. It also lets users prove who they are and securely sign transactions. Hash functions are used to give each piece of data a unique identifier. This keeps the information on the blockchain secure.
- Access Control and Privacy: Access control mechanisms can be used to control who can use the blockchain and the smart contracts that go with it. Different people in the system have different permissions and roles, which are set by these mechanisms. This gives the user fine-grained control over who can see and change data. In addition, techniques such as “zero-knowledge proofs” or “privacy-preserving algorithms” can be used to improve privacy and make sure sensitive information stays private while allowing for secure interactions.
- Encrypted Data: The system can use encryption techniques to keep sensitive data, such as personal information or details about energy use, private. Data that have been encrypted can be stored on the blockchain or in storage that is not part of the blockchain. This way, authorized parties who have the decryption keys can safely access and use the information.

By using these parts of blockchain technology, our system makes sure that data and transactions are secure, private, and correct. The distributed ledger, immutability, consensus mechanisms, cryptographic security, access control, privacy measures, and encrypted data all work together to keep the system safe from unauthorized access, tampering, and data breaches.

### 3.4. Blockchain-Based Setup and Execution

This study utilized Ganache for creating a private Ethereum blockchain. It was utilized to test the solidity contracts. Contrary to Remix, it offers a higher number of better features. We installed the Ganache from its official website and linked it with VsCode. The home screen is what the user sees when they first launch Ganache. The user is given the choice to load an existing workspace (if one already exists), start a brand-new custom workspace, or quickly launch a one-click blockchain with the default settings on this screen. In the screenshot, two user accounts are displayed. Their combined balance is ETH 100 where Ether is a currency used for transactions on the Ethereum platform. Additionally, the total of each account shows 0, indicating that the user did not complete any transactions yet.

The user chooses “Deploy Contracts” from the context menu when they right-click on the smart contract file while Ganache is executing. This displays every available network, including networks that the user has set up with the extension and those from their truffle-config file (Infura and Ganache). The extension deploys the user’s contract to Ganache when they have chosen the Ganache network.

**Figure 9:**
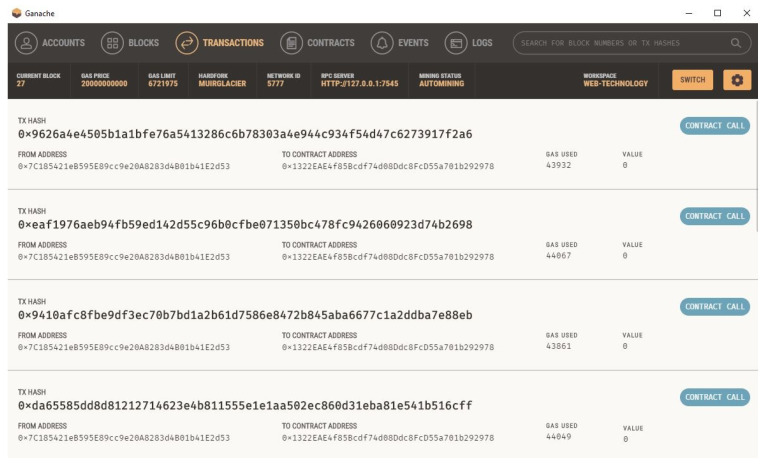
Solidity blockchain smart contract (VSCode).

**Figure 10:**
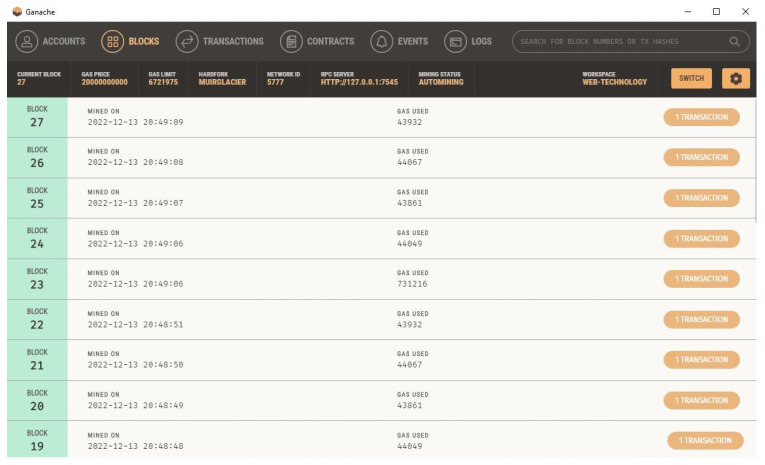
Ganache blockchain listings after the smart contract.

This addon offers a unified interface for everything related to smart contracts. The outcome of our smart energy contract deployed in the Ganache VSCode environment is shown in Figure 9 and Figure 10.

## 4. Results

Many performance evaluation metrics are used in the literature for prediction models. We used the moving average as our baseline model for time series forecasting for smart home energy usage under different weather stats. The moving average is mathematically represented as Equation (1), which gives a threshold for beating the root mean square error (RMSE).

(1) $$\left(y_{t}\right)=\frac{1}{n}\sum_{i=1}^{n}\left(y_{t}-i\right)$$

**Figure 11:**
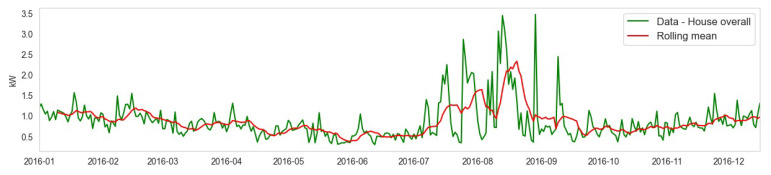
Moving average of the house overall data is plotted in green lines from January 2016 to December 2016, on a monthly basis is represented in blue line, and the rolling mean that is applied on it is represented by the red line.

We plot the ’House overall’ energy usage and its rolling mean in Figure 11.

**Figure 12:**
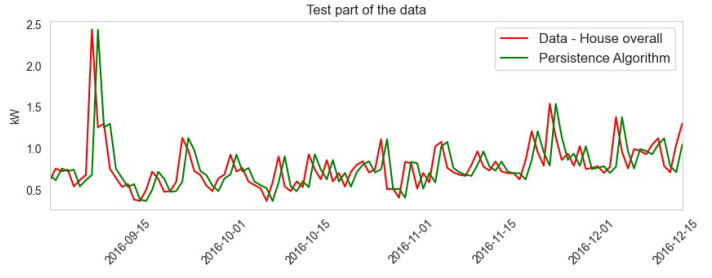
Results of the ARIMA model.

The moving average of the house’s overall data is plotted in blue lines from January 2016 to December 2016 on a monthly basis. The rolling mean is applied to it and is shown in the red line. It gives trends of energy usage over short periods using a set of data on a monthly basis. From the figure, it can be observed that the prediction is not very accurate as it considers short-term data only. Afterward, ARIMA was applied to the dataset. ARIMA utilizes past values to work on a given time series data. The results are presented in Figure 12.

The ARIMA model prediction is shown in red lines. The dataset was split into 70% training and 30% test data in which January to September training data are shown in green lines and September to December are test data shown in blue lines. The outcome shows that the maximum prediction is accurate.

**Figure 13:**
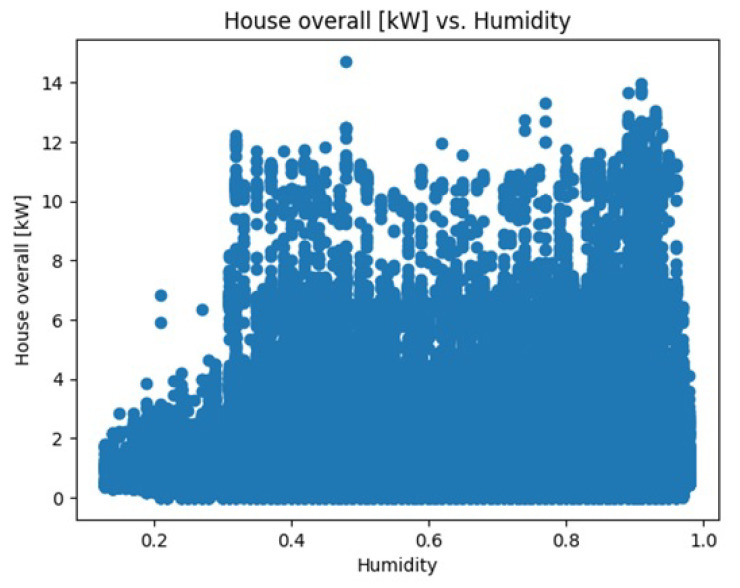
Scatter plot of ’House overall [kW]’ variable with different levels of humidity.

Figure 13 represents a scatter plot showing how the ’House overall [kW]’ variable changes with different levels of humidity.

The dataset contains information about the electricity usage of a home over time. We considered three attributes—’temperature’, ’humidity’, and ’pressure’—which were used as inputs to create a model that can predict the ’House overall [kW]’ column in the dataset to represent the total electricity usage of the home. By using linear regression, we presented a mathematical relationship between these input variables (temperature, humidity, and pressure) and the output variable (total electricity usage). The performance of the model was evaluated by comparing the predicted values to the actual values using scatter plots and histograms. This allows us to see how well the model fits the data and identify any patterns or issues that may be present. The R-squared value is 0.0004638 and the predicted electricity usage is 4.80019.

**Figure 14:**
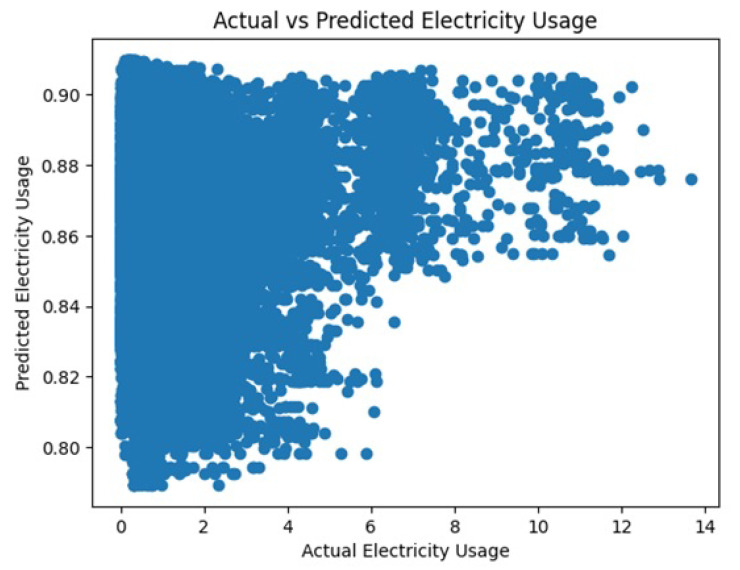
Scatter plot of actual vs. predicted electricity usage.

**Figure 15:**
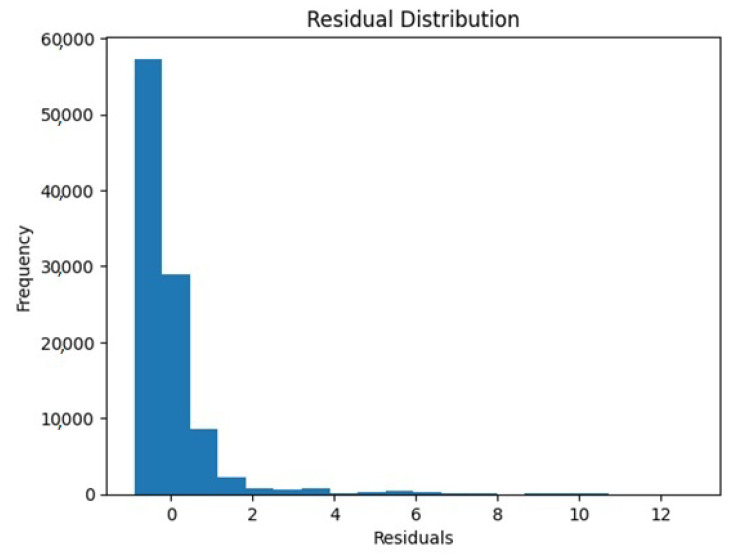
Residual distribution.

Figure 14 and Figure 15 represent the actual vs. predicted usage of electricity and residual distribution, respectively.

We implemented a long short-term memory (LSTM) neural network to predict energy consumption using historical energy consumption data against smart home datasets.

**Figure 16:**
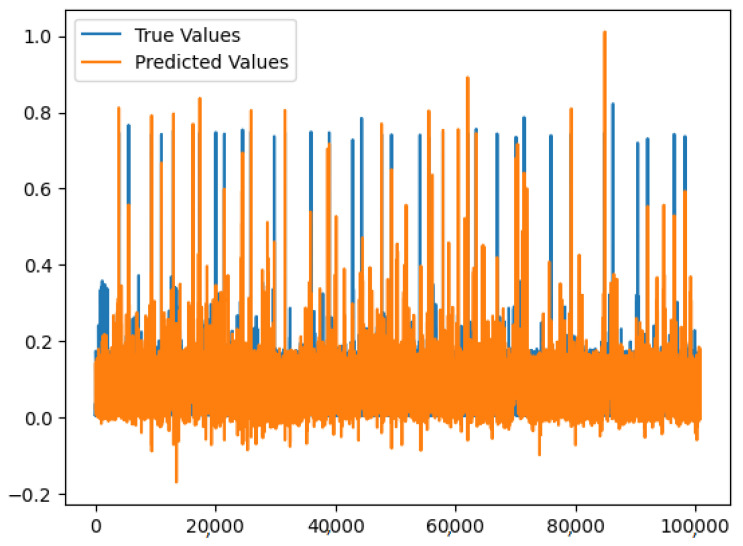
LSTM results on the dataset.

Figure 16 shows the predicted value of energy consumption from the model based on the input data from the test dataset. The accuracy score of the model is correct 99.52% of the time when predicting the energy consumption values. The model predicted a value above a certain threshold, but only 75.32% of those predictions were actually correct.

### 4.1. Formal Approaches for System Validation

Formal methods validate AI-based methodologies. We can evaluate our system’s behavior and performance using mathematical models, logic, and formal approaches. This method detects system faults, biases, and vulnerabilities. Safety, security, robustness, and fairness can be verified using formal methods. To guarantee that the proposed system fulfills standards, we can formalize and evaluate these attributes using formal specification languages and model-checking methodologies. Formal verification helps to uncover property discrepancies and allows for corrective action. It can identify and address system behavior risks, uncertainties, and unintended effects. This gives end-users, regulators, and industry professionals confidence in our suggested approach’s reliability and safety. A number of studies [44,45] have described formal approaches for AI and ML-based systems. Acknowledging the importance of formal approaches, we will incorporate formal verification techniques with our proposed system in the future.

### 4.2. Overhead of Proposed System

Blockchain technology requires each node to store a distributed ledger of transactions or smart contracts. As blockchain data grow, so does storage. For prediction, the system may save historical energy usage data, which increases storage overhead. Blockchain systems propagate and validate transactions through a network of nodes. Communication overhead increases network traffic, latency, and bandwidth. Edge computing reduces network overhead by calculating locally, but data synchronization and consensus need node communication.

Scalability issues arise when participants or transactions increase in blockchain systems. As the number of smart houses and devices in the monitoring system rises, the blockchain network may encounter transaction processing and validation delays, reducing performance. Building and integrating a blockchain-based smart home monitoring system with energy usage prediction involves expertise in blockchain technology, edge computing, predictive modeling, and smart homes. Complexity can add time, effort, and expense to the system design and implementation.

Implementation specifics, design choices, and the system scale can affect overheads. Planning, resource allocation, and optimization can reduce these overheads and increase the system performance and efficiency. We intend to analyze these overheads in the future

## 5. Conclusions

A blockchain-based secure IoT smart home system was proposed and machine-learning-based energy demand prediction was presented in this work. We utilized the dataset of energy use in a smart home with one year of data logging in various weather conditions. We used the statistical models ARIMA and moving average, and we compared the outcomes to the LSTM, which is based on deep learning. The outcomes demonstrated that the LSTM model performs more effectively. Additionally, the framework adds security, confidentiality, and integrity considerations to the proposed system using blockchain technology. To validate the security of the proposed framework, it can be used to create Ethereum-based net metering in a practical setting. In the future, we aim to analyze the proposed model with respect to the system setup, data storage, and computing efficiency overhead. We also aim to conduct a formal analysis to achieve system validation.

## Figures and Tables

**Table 1:** Comparative analysis of discussed research works.

Ref.	Description/Goal	IoT Platform	Smart Home Energy	Blockchain Platform	Security
[9]	Examined the various smart home modules, their functions, and the corresponding transactional techniques.	Smart home	No	Yes	N/A
[15]	Presented a smart-contract-based IoT architecture comprising device and server architecture on Ethereum that allows for better security against DDoS and rogue device attacks.	No	No	Etherium	-
[30]	Reviewed, classified, and assessed solutions for DDoS attacks. Solutions based on a distributed architecture, access management, traffic control, and the Ethereum platform were investigated.	No	No	No	No
[31]	Presented an algorithm for a recommender system for energy saving in smart homes through mining consumer behavior data.	Home automation system	Yes	No	No
[33]	Put forth a lightweight hash-based blockchain design that can adjust the mining hashing algorithm to network traffic.	IIoT	No	Yes	Quark, Photon, Spongen
[32]	Analysis and assessment of lightweight hash algorithms for IoT devices based on blockchain	Yes	No	Yes	RSA, SHA, Keccak, AES
[34]	Explored private Ethereum blockchain concerning the smart contract.	Yes	No	Ethereum	RSA, SHA, AES
[1]	Investigated the volatility of energy prices and PV generation. Proposed robust hybrid stochastic optimization model. Energy management in real-time.	Smart Home	Yes	No	No
[35]	Analysis of recent developments and discussion on using blockchain in smart homes.	Smart home	No	No	No
[36]	Discussed IoE with blockchain	No	No	No	No
[37]	Secure and efficient smart home architecture	Yes	No	Yes	Yes
[12]	Addressed the difficulties, opportunities, and potential solutions for integrating blockchain and IoT into power networks for managing the supply of home electric vehicles.	Yes	No	Yes	No
[3]	Secure smart home systems using a blockchain-based approach.	Yes	No	Yes	Yes
[38]	Proposed a solution for trading energy without a middleman. The solution is a blockchain-based LEM model.	Yes	No	Yes	Yes
[39]	Investigated a privacy protection scheme for a smart home.	Yes	No	Yes	Yes
[40]	Investigated HEMS for energy usage reductions. The focus is on a population segment of particular relevance.	Yes	No	Yes	No
[16]	The performance of HEMS with RTP using meta-heuristic optimization techniques	Yes	No	No	No
[41]	Examined many philosophical and technical aspects of effective power management at home front	Yes	No	No	No
[42]	Step in organizing the research operations in SHEM was to create a pyramid with four functional layers: monitoring, analyzing and forecasting, scheduling, and coordinating.	Yes	No	No	No

## Data Availability

The data used in this study is available from authors on reasonable request.
